# Is metabolic syndrome a modifiable risk factor for gout?

**Published:** 2023-05

**MAY 3, 2023 -** Short-terms clinical trials have demonstrated the health benefits of low-carbohydrate diets (LCDs) and low-fat diets (LFDs) for weight loss and heart protection. Now a study published in the Journal of Internal Medicine looks at the effects of these diets on mortality in middle-aged and older adults.

In the study of 371,159 individuals aged 50 to 71 years, 165,698 deaths occurred over a median follow-up of 23.5 years.

A healthy LFD—characterized by low intake of saturated fat and high intakes of plant protein and high-quality carbohydrates—was related to fewer deaths from all causes, from cardiovascular diseases, and from cancers. In contrast, an overall LCD and an unhealthy LCD were associated with significantly higher total, cardiovascular, and cancer mortality rates. A healthy LCD was associated with slightly lower death rates.

“Our results support the importance of maintaining a healthy LFD with less saturated fat in preventing all-cause and cause-specific mortality among middle-aged and older people,” the authors wrote.

***URL upon publication: https://onlinelibrary.wiley.com/doi/10.1111/joim.13639***

***Full citation:** “Low-carbohydrate diets, low-fat diets, and mortality in middle-aged and older people: A prospective cohort study.” Yimin Zhao, Yueying Li, Wenxiu Wang, Zimin Song, Zhenhuang Zhuang, Duo Li, Lu Qi, Tao Huang. J Intern Med; Published Online: 3 May 2023 (DOI: 10.1111/joim.13639).*

*Copyright © 2019 The Cochrane Collaboration. Published by John Wiley & Sons, Ltd., reproduced with permission.*

